# Laboratory animal strain mobilities: handling with care for animal sentience and biosecurity

**DOI:** 10.1007/s40656-022-00510-1

**Published:** 2022-06-29

**Authors:** Sara Peres, Emma Roe

**Affiliations:** School of Geography and Environmental Science, University of Southampton, Southampton, UK

## Abstract

The global distribution of laboratory mouse strains is valued for ensuring the continuity, validity and accessibility of model organisms. Mouse strains are therefore assumed mobile and able to travel. We draw on the concept of ‘animal mobilities’ (Hodgetts and Lorimer 2019) to explain how attending to laboratory mice as living animal, commodity and scientific tool is shaping how they are transported through contemporary scientific infrastructures and communities. Our paper is framed around exploring how animal *strains* travel, rather than animals, as we show that it is only through understanding strain mobility that we can explain how and why live animal movement can be replaced by germinal products. The research is based on qualitative fieldwork in 2018 and 2019 that included 2 weeks ethnography and interviews with key informants involved in the movement of laboratory animals. The empirical analysis discusses practices that relate to managing biosecurity and animal welfare concerns when moving laboratory animal strains. In closing we reflect more broadly on the contemporary ‘ethico-onto-epistemological’ (Barad, 2014) entanglement that shapes who or what travels to support laboratory science data-making practices, and the intensity of care ‘tinkering’ practices (Mol and Law 2010) that facilitate the movement. We explain how a laboratory animal strain exceeds its value solely as a mobile and thus exchangeable commodity, illustrated in how values that relate to animal sentience and infection-risk supports its material transformation. Consequently, it is becoming increasingly common for non-sentient germinal products – embryos and gametes - to replace live sentient animals when being moved.

## Introduction

The global distribution of laboratory mouse strains is valued for ensuring the continuity, validity and accessibility of biomedical research that use model animal organisms, nationally and internationally (Kenyon & Fray, 2017, p. 386). Mouse strains are therefore expected to be mobile and to travel: indeed, moving them between institutions, both within and between countries, is a normal procurement practice in the contemporary landscape of obtaining animals for use in animal research. The evidence for this is the existence of large repositories such as the Jackson Laboratory and the European Mouse Mutant Archive that act as repositories and distributors of laboratory mouse strains, as well as countless scientific collaborations where researchers share access to existing models. Each animal becomes categorised as a living embodiment of one of a huge variety of laboratory animal strains, which in turn are considered highly-specialised scientific tools (Kohler, 1994; Clark, 2014) used in support of the generation and circulation of knowledge as model organisms (Nelson, 2018) and the accumulation of capital.

Since strains are being moved as a tool and exchange-commodity within the scientific industry of knowledge-making, there are concerns surrounding how the mouse strain journey might threaten the delivery of reliable scientific data outcomes. A journey unsettles values and experiences of consistency achieved through stasis; consequent changes to materiality and meanings become attached to the mobile form of the living strain, furthering its instability through now meaning as well as its lived experience. We describe how laboratory animal strains in fleshy, warm form as a living animal or as a frozen animal body-part or body-product when mobile, achieves value as a commodity form, but never as a stand-alone, alien capitalised objects (Tsing, 2015) so complexly are the value-relations that build around them.

We draw on the concept of ‘animal mobilities’ (Hodgetts and Lorimer 2019), to address the implications for laboratory animal strain mobility from the multiple material value cultures of laboratory mice - as living sentient animal, commodity and scientific tool or resource. This idea has two inferences, which the authors distinguish with an apostrophe (Hodgetts and Lorimer, 2019). Firstly, animal mobilities refer to “the movement (or stillness) of animals as shaped by the actions of various actors, particularly humans”. However, secondly, there is a ‘tactical distinction’ in studying animals’ mobilities, which Hodgetts and Lorimer describe as “the embodied, affective, and lived animal experience of mobilities” (Hodgetts and Lorimer, 2019: 5). In using the lens of animal mobilities we look into the tensions between the animal strain as an exchangeable and commodified research tool, and the live animal’s experiences of travel that can damage the commodity; innovations to tackle this are having wider implications on the scientific infrastructure. Our study is set within the contemporary scientific procurement infrastructures and communities of the UK research industry.

We frame the paper around exploring how animal strains travel, rather than animals. This builds on Davies’ suggestion that mice are easily “displace[d] (…) to the genetic identity of the inbred strain’ (Davies, 2013, 132), as we show that it is only through understanding strain mobility that we can examine how and why sentient animals, and animal body parts, move across space within research animal procurement processes. In this way we bring a feminist materialist concern to flesh-out understanding of laboratory animal strain journeys. We present findings, that show that culturally-situated concerns for infection risk as biosecurity, sentient animal welfare and reliable scientific knowledge-production assemble to shape how a mobile commodified laboratory animal strain can change form as it makes a journey between origin and destination. This change of form refers to the replacement of live sentient animal transport with non-living alternatives such as non-sentient, latent embryos or gametes rather than as sentient animals, as has been discussed in the case of genetically modified animals (Robinson et al., 2003; Swallow et al., 2005). This increasingly common replacement of transporting live sentient animals with non-sentient germinal products, where then a surrogate mother is used to carry the implanted embryo till birth and so the strain is rederived, indicates ongoing and future changes to the ‘ethico-onto-epistemological’ entanglement (Barad, 2014) when strains move and its relation to scientific knowledge-production and standardising the research tool. What values found within the animal research industry is shaping this contemporary ethico-onto-epistemological entanglement of laboratory animal strain mobility?

This work draws on longitudinal ethnographic research over 2 separate weeks, one with a UK research institution with a biobank, and one with a UK team of people involved in the breeding and procurement of animals for research. The ethnography enabled close study of how laboratory animal strains move both as living animal and the process of their movement as rederived germinal products. In addition to the ethnography, 23 in-depth semi-structured interviews were made with key informants in the supply chains of laboratory animals, and various site visits.^1^ The key informants included facility managers, researchers, biobank technicians, laboratory animal technicians, animal research administrators and breeders. These interviews and the ethnography were undertaken between 2018 and 2019 by researcher Peres. Researcher Roe has previously carried out two weeks ethnography and many research facility site visits over the last 8 years, which has also informed the interpretation of the new data. Throughout the fieldwork we sought to understand the practices, feelings and opinions that help explain how and why research animals move and how biotechnological developments. such as biobanking and germinal product transfer and strain resurrection through rederivation, are becoming more popular as an alternative option to live animals. Much of this work is performed by animal care technicians who hold an important practical role in preparing and receiving transported animal strains (Greenhough & Roe, 2017) and so they are an important voice in our study.

Within the UK, where this study is located, some of the regulation of animal mobility is articulated by guidelines that set out the requirements for humane animal transportation (National Research Council, 2006; Swallow et al., 2005), whilst others are the consequence of other localised logistical demands, and biosecurity factors. We show that live sentient animals and their non-sentient, non-living embryos and gametes have different mobilities, which are shaped by differing regulatory, ethical and scientific values attached to sentience and biosecurity. This contributes to work within animal studies where the material biopolitics of animal sentience and biosecurity is driving a question about not only the acceptability of how research animals are used by humans (Asdal et al., 2017), but also their suitability as animal health and welfare concerns increase in dominance, especially in relation to laboratory animal strain mobilities. We go on to illustrate how the mobilities of laboratory animal strains are regulated and implemented with a view to minimising welfare issues and carefully addressing biosecurity, whilst enabling the movement of animals to take place.

In the next section we discuss the contemporary regulatory, industrial and wider societal concerns as the context that informs laboratory mice movement.

## Context: regulation, scale of laboratory mice movement and social controversy around animal research

Within the UK, where the study is located, most laboratory animals will move at some point in their lives, but their journey-length can be as short as between adjacent rooms within the same facility, or as long as thousands of miles, lasting several days^2^. Perhaps given this diversity, it is difficult to quantify even the number of lab animal journeys by road, air or sea within the UK alone. However, Home Office annual statistics suggest that at least 1.9% of laboratory animals used in procedures in the UK were imported into the country (Home Office 2019, p. data Table 2.1). Similarly, the numbers of animals bred in the UK for research and then exported are not freely available.

Despite the regular movement of laboratory animals around the country, they are far less visible than other animals on the move. For instance, laboratory mice travel in unmarked air-conditioned, windowless vans, in contrast to open-slatted livestock trucks visible on the road network. Indeed, where the transport of research animals has become public knowledge, it has also become a pressure point for activists (McAdams et al., 2017, p. 38) through campaigns such as “Gateway to Hell” (now apparently inactive) which called on several airlines to stop transporting animals^3^. Since then, some leading air carriers and ferry companies refuse to transport research animals (Clark 2005; Pain 2016). However, the reduction in available carriers can lead to longer journey-time or involve less than ideal transport options, such as taking indirect flights. Nevertheless, the double hit of public concerns about laboratory animal research combined with wider concern about animal transportation has led to corporate transport actors restricting the mobilities of laboratory animals. Animal transport, then, may invoke “societal sentience” (Hobson-West & Davies, 2018): affective responses from publics that, in turn, have practical and affective implications for everyday animal transport practice. Cresswell writes, in reference to outbreaks of zoonotic and livestock diseases, that “[m]any of the moral panics about animals have had mobility at their heart” (Cresswell, 2014, p. 715). Indeed Wood’s (2012) shows historically how concern for animals’ mobilities was an important factor in the establishment of animal welfare as “a term, a concept and a target of government regulation”, when the British government sought to regulate animal transit in the 1960s and 1970s. We can see from this how political and social commitments to address animal welfare concerns closely influence the regulatory/administrative apparatus to enable or block animal (livestock) trade or transport (Kirchhelle, 2021).

Commodified animal mobilities have been bound up with trading and exchanging animals in global capitalist economies for many years. They have also been the focus of international political and biosecurity disagreements, for example between British and European identities with respect to policies for transported animals (Message, 2019). Franklin’s (2007) narrative account points to the significant role that regulation plays in constraining the movement of animals; for instance, a flock of sick sheep unable to return to Australia aboard the *Cormo Express* ship because of biosecurity regulations, and thus stuck at sea after an outbreak was found aboard the Saudi Arabia-bound ship). In this example it is biosecurity laws with requirements for health certification for animal consignments as ‘commodities’^4^ and import/export paperwork that is impinging on the easy movement of animals across borders. Contrastingly, Law (2006) draws on concepts from fluid engineering to describe the engineering of flows, barriers (keeping viruses and animals apart) and exchanges (interactions between vaccinated animals and the virus) in contemporary industrialised agriculture, during the UK Foot and Mouth Disease crisis in 2001.

Until recently, and during the period of our data collection, the legal framework regulating the import and export of laboratory animals originated in European law. The European Council’s Balai Council Directive 92/65/EEC sets out the rules with respect to animal health for transport within the EU, for live animals as well as sperm, eggs and embryos for research or conservation (and commercial pet animal movement)^5^. Balai mandates the need for animal health certification to travel with every live animal and germplasm^6^; this certification is validated by an official veterinarian at the source and the information was, until recently, uploaded to the EU’s Trade Control and Expert System (TRACES) at least 24 h prior to despatch. This allowed the waiver of immediate border controls since the British Animal & Plant Health Agency (APHA) were able instead to monitor it virtually or through checks at the animals’ final destination.^7^

Research animal imports and exports are not, of course, limited to the European continent. There have for a long time been additional requirements for non-EU destinations and, at the time of writing, the governance framework is changing due to the UK’s exit from the European Union. The Balai rules were incorporated into UK domestic law as ‘retained EU law’, so that the process remained nearly identical in the months following Britain’s exit from the EU. However, from March 2022, Balai animals’ consignments are no longer moved seamlessly into the United Kingdom. Instead, they may only be imported into the UK through ‘an approved Border Control Post’ (Animal and Plant Health Agency 2020: 6) where checks of the documents or the animals themselves may take place. The post-import checks of animal health certification, then, are an administrative process to manage biosecurity risk attached to animal transport. Its presence has implications on processes of decision-making about how to move an animal strain given the need to administrate and assure biosecurity.

Within animal welfare science studies there is a large and varied species-specific literature to measure the effects of ground (e.g. Beagle dogs - Herbel et al., 2020), air (e.g. red king crab - Mota et al., 2021) and sea transportation (e.g. Sheep - Willis et al., 2021) of animals, and even space travel for mice (Choi et al., 2020). When compared to other industrial sectors that transport animals, the animal welfare literature for the transportation of laboratory animals is small (but see Swallow et al., 2005; Claassen, 1994). Recent research efforts to provide more detail on the stress an animal experiences during transport have identified a variety of relevant social, environmental and biophysical factors, as illustrated by this quote:

During transportation and upon receipt, they [mice] are exposed further to a different environment, which may include vibrations, sounds, disruption of the dark/light regime, temperature, humidity, feed, bedding, water, odors, unfamiliar animal caretakers, and a new social structure. These factors may induce stress as a cumulative burden, possibly affecting animal welfare and confounding research results. (Rumpel et al. 2019:2)

Consequentially, research has shown changes in the transported animals’ physiological parameters such as heart rate, breathing rate, blood pressure, body temperature and hormone levels (Arts et al., 2014; Rumpel et al., 2019), based on their recent research, go on to offer a general guide that:

At least seven days of acclimatization is recommended following transport between sites and at least three days between buildings on the same site [22]. However, depending on the severity and duration of the stress experienced and the parameter to be investigated, the period of acclimatization may take several weeks to normalize, as observed for blood pressure (three weeks) [23], as well as corticosterone and immune parameters (three weeks) [24] in BALB/c mice. (ibid: 8)

Moving animals for research, then, involves challenges to animals’ welfare and poses risks to their reliability as data sources after arrival. In recognition of the effects of travel on animals, there are national and international regulations that specifically address the animal’s welfare during transport, via land, sea, or air. 8^,^^9^ To support these legal mandates there is specific guidance on animal movement outlined in the Guidance on the Operation of Animals in Science Procedures Act (Home Office, 2014). Various organisations, such as the Laboratory Animals Science Association, have published supplementary regulatory guidelines in the UK, which emphasise the need to ensure legal compliance and adoption of best practice with regards to animal transport, with a view to “significantly improve the welfare of laboratory animals undergoing transportation” (Swallow et al., 2005, p. 5). Collectively, this regulatory framework involves considerable administrative requirements and can be rather costly. How have social science and humanities scholars of laboratory animal research studied the movement of laboratory animals and the scientific infrastructure that surrounds and drives it? And, to what extent have they considered findings of animal welfare science in relation to animal’s mobilities? How can studies of the industry look beyond a capital-centric explanation to the practices of handling mice on the move to show how value norms and practices articulate ethical concerns both internally and externally?

## Literature review

Scholarly writing on laboratory animal journeys within a globalised scientific economy feature in historical accounts, for example the animal transport practices instigated by the establishment of centres of breeding expertise like the Jackson laboratory (Rader, 2004) or the Medical Research Council’s Laboratory Animals Bureau (Kirk, 2010). Animal journeys are intrinsic to the process of breeding and distributing standardised animals for science. It is also critical to protecting against genetic drift of colonies by enabling access to the progenitors needed to breed animals of known genetic constitution. Within contemporary studies there is an absence of literature on the everyday practices enabling strain mobility, or how these are shaped and shaping global and local commercial and scientific infrastructures.

Davies (2013) indirectly refers to these laboratory mouse journeys when she details them as, “becoming worldly”, as “experimental organisms developed in one laboratory become standardized technical commodities, spreading from individual research centres, through specialized laboratory suppliers, to become the patented property of international biotechnology” (ibid, 131). She found, as part of her biogeographical exploration of transgenic mice, that in the post-genomic era it is not only standardisation, but also the generation of biological difference that can generate novel scientific and economic value. Another way that place of origin matters in different ways at the destination is through the increasing recognition of place of origin mattering in determining identity. The ubiquitous, standardised C57 Black 6 strain, for instance, is “not everywhere and always the same” (Davies, 2013, p. 146): each substrain has final letters on its code to “acknowledge the originary points of these universalised strains” (Davies, 2013, 146); the Taconic Black 6 is not the same as the Jax Black 6.

One of the implications of this ongoing recognition and relevance of geographical origin is that mouse strains must become mobile to continue lines of research using specific models. Because of this awareness, “new sites of biomedical research (…) find themselves locked into the use of these standardised but also mutable animals, their animal houses needing constant resupply from (…) established stock, historically situated in Europe or the USA, if their research findings are to find a place in international journals” (Davies, 2013, 146). Consequently, the mobility of laboratory animals is closely linked to scientific value-making practices through the dissemination of potentially valuable biological difference, that of animals needed to maintain consistent research model standards through careful colony management practices.

Another contemporary development in scientific culture is growing cooperation by the academic scientific community to create a “mouse academic [research] commons”^10^ (Einhorn & Heimes, 2009). This is a vision of the widespread availability of mice strains with no substantive restrictions to their use, which requires, in turn, that they travel. For Parry, these are understood as “open source economies” where “[e]ngineered mouse strains are (…) best conceived of as an experimental space or biological commons open to re-invention by all” (Parry, 2019, 1). Within the social scientific literature that critically examines the organisation of this commons (Bubela et al., 2015; Mishra et al., 2016), we are interested in how community sharing and property becomes practiced. Material Transfer Agreements that accompany animals as they move are part of the changing scientific infrastructure, in how they stipulate legal expectations that shape how mobile laboratory animal strains are shared, very often with stipulations about what receivers can and cannot do with animals, and possible conditions for any future movement of the strain.

### Lab animal strain mobilities

The concept of ‘animal mobilities’ sits at the intersection of animal geographies (Buller, 2012) and mobility studies (e.g. Cresswell 2011a; 2011b; 2014). There are different inferences to the use of this term that are relevant to how one might draw on this approach to study laboratory animal mobilities. Mobility, as Cresswell describes,

is as much about meaning as it is about mappable and calculable movement. It is an ethical and political issue as much as a utilitarian and practical one. (Cresswell 2011a, 552)

The lens of animal mobilities thus encourages analysis beyond the mapping of where, how far, or how many laboratory animals move between places. Instead, practices and meanings – globalised science, mouse commons, public concern about laboratory animal transport and others - as they relate to how mobility structures human-animal relations become the centre of analysis. Whilst human-human relationships between different nations, institutions, different parts of the research animal supply chain - breeders, buyers, researchers, animal technicians, biobank technicians, administrator – configure aspects of research animal mobilities, folding in the agentive material capacities of the materially shape-shifting animal strain as living animal, embryo or gamete into the analysis, provides different insights to laboratory animal strain mobilities and their place in scientific knowledge-making industry. Laboratory animal strain mobilities are co-constituted through “movements [that] are always produced within (and are productive of) relations of power between various actors” (Hodgetts & Lorimer, 2020, 5) and our analysis includes within the category of actor the agentive materiality of the laboratory animal strain itself whether in sentient or non-sentient form. If the lived experience of a sentient animal or their non-sentient fleshy products, are not acknowledged as holding powers to shape, to co-constitute what is going on, but to instead presume humans hold all the power, our analysis will be insensitive to how the material world shapes what humans do and think. It will be all the poorer for it and show no generosity in making space for animals and other non-humans as political actors that humans have always been generating knowledges with, and not from. And yet, how we generate knowledges with them is constantly changing, as illustrated through our findings in this paper.

## Mobilising strains: empirical findings

### Live animals’ mobilities

When animals move into and out of different institutions, each journey requires careful planning and coordination. While researchers may request the purchases or negotiate the sourcing of animals from collaborators, the move is generally organised by animal care staff, sometimes in liaison with administrative staff. Indeed, there is a distribution of labour involved, where researchers may be somewhat less aware of the logistical and surveillance requirements involved in making animals mobile, to the frustration of animal care staff.^11^ Checks must be performed, and licences signed, before any movement can take place. Veterinarians (in UK laboratories, the Named Veterinary Surgeon) take legal responsibility for assuring that the animals are ready for travel.

The mobilities of laboratory mice are facilitated by practices like cage cleaning, quarantining, specialised animal courier services, animal care at departure and arrival, and discretion. Animal care technicians (ATs) do the hands-on work of packaging and unpackaging the animals at their departure and destination, and ensure that the animals appear in good health, have sufficient food, water, and bedding. To ensure some sense of familiarity as they encounter the changing world of being on the move, ATs settle mice into their travelling box some hours before they are due to leave^12^.

There are certain expectations surrounding the distribution of responsibilities and actions of suppliers and couriers in the movement of animals. Namely, receiving animal care staff have care and health expectations. For instance, Jemma notes that they “*would expect every animal to be health assessed, not boxed up too early and have, you know, the gel packs and the food that they require with bedding as well*.” (Jemma, Named Animal Care and Welfare Officer, University). Discretion surrounding the movement of live laboratory animal mobilities is also desired. Exposure of the practice to an outsider’s eye can cause apprehension for staff members involved in the shipping and receipt of animals. As a result, steps are taken to avoid attracting attention. For example, in one of our ethnographic sites, animal purchases were categorised as “miscellaneous” on the University procurement system to avoid attracting attention, and technicians were not comfortable with the placement of the loading bay, considered a possible risk as too visible from the street.

Moving animals is a complex event. The following extract from fieldwork notes describes part of a journey where two animal technicians, Lyndsey and Ida, are bringing boxes of mice from one facility into another, that happen to be at the same institution, and thus geographically close. This quote illustrates two main concerns: on the one hand, the cleaning practices and the apparel involved illustrate the need to minimise infection risk: fresh Personal and Protective Equipment (PPE) clothing, the meticulous cleaning, the use of airflow to reduce any ‘contaminants’ that are in transit with the cage, or from the outside world. On the other, the very process of travelling has implications for the welfare of the mouse passenger within.

*’Lyndsey puts on extra white overalls, white overshoes, a hair net on top of her existing scrubs and blue overall to meet the mouse traveller. She goes and waits at the exterior, dirty side of the port. Behind her are transparent stiff plastic curtains. Ida gets into the interior, clean side of the hatch. Air flows through the hatch from clean to dirty to restrict dirty air from moving into the clean lab space.*

*Working quickly, Lyndsey takes the first IVC [individually ventilated cage] of mice and sprays the exterior with disinfectant and then wipes it down top and sides. Finally, carefully lifting it up to wipe the base with disinfectant. Then she opens the sliding window that separates the two sections of the port and passes the cage through to Ida, who stands on the facility side. Ida then repeats the same cleaning process, top, sides and bottom with disinfectant, before placing it on a black trolley in the room.*

*Finally, my attention turns from the cage to the passenger inside, a white mouse pirouetting inside. Puzzled by what this movement means, I ask Ida who looks at it attentively. She explains this behaviour can happen when mice get stressed from travelling and that she will keep an eye on this mouse.’ (Peres, fieldnotes)*

This extract illustrates the level of practical attention to biosecurity and animal welfare during this animal journey. In the next section we develop our discussion of these key influences on how laboratory animal strains travel, in greater depth.

#### Health status report

Live animal mobilities are shaped by concern about possible disease transmission associated with animal movement. If mistakes are made, and disease enters the lab, this can lead to the mass-culling of animals, loss of data and delays to experiments. Barrier facilities operate to prevent pathogen contamination, and also meet scientific demand for animals with a particular microbiological status – such as the germ free animal (Kirk, 2012). Consequently, any movement of mice into a facility begins with the transfer of documents that can be applicable to an individual or a whole group of animals, as they move through laboratory mice supply chains. Specifically, veterinarians will check for the risk of disease transfer in a health status report, known as health screening. Since “health screening” is an essential step in determining whether animals can enter a facility or not (further quarantining notwithstanding), the mobility of a mouse depends on the perceived biosecurity status of their place of origin, and the ability to demonstrate certain biomaterial realities “on paper’. The health status report is a critical piece of travel documentation that dictates the mobility of mouse strains, although it is more akin to a permit to travel to a destination, and cannot be considered in all cases to be the ‘market instrument’ that Enticott (2016) describes in relation to farm animal movement, due to the under-developed market around many types of research animals.

The biosecurity reputation of the institution of origin is an important factor in determining the future movement of mice. Approved suppliers with whom a facility has a longstanding agreement, are deemed to be of sufficient quality that the animals can enter because of the assurance provided by their institutional health certification. For instance, Leonard, who heads a university biological services facility, frames it in terms of trust:

*If we’re bringing live mice into the facility, we have a list of approved suppliers [by] unit managers and the Named Veterinary Surgeon, [stating] that we trust them as a source and their health status is acceptable to us. It includes the major commercial suppliers and a handful of other institutes. (Leonard, Facilities leadership, University)*

The mobility of laboratory animals, then, depends on assessment of the risk posed by them as vectors of infection. Not all sources of laboratory mice are understood to have the same levels of risk: facility staff judge the likelihood of infection depending on the animals’ origins, and the strength of its surveillance system. The mobility of live animals is continually shaped by the biosecurity risk that they pose. The “trustworthiness” of suppliers is therefore firmly brought into contractual responsibility to diligently mitigate against infection spread, or what Barker (2010) would describe as ‘biosecurity citizenship’ to act on the ‘dangerous’ biological mobility potential hidden on a seemingly healthy mouse. A trustworthy supplier can demonstrate that they monitor their colonies for infection and can act on that knowledge. Yet, as the quote below demonstrates, surveillance per se isn’t necessarily sufficient to control infections and foster assurance; there are sometimes mismatched expectations about what it means to act appropriately.

*We have a supplier that we once bought animals from and they [previously] brought in norovirus and (…) I found out afterwards it was reported, but it wasn’t made really very obvious. I would never have brought them in had I known. I’ve learnt since then to look at lot more carefully at the screening reports, etc. [laughs]. But you know, it should have been in Big Red Writing, I feel. (Jeremy, Operations leadership, University)*

The journey is also very directly defined by biosecurity concerns in the sense that animals can travel through quarantine spaces before they are cleared to enter some facilities. In the example below, a Named Animal Care and Welfare Officer (NACWO) working at a university-based transgenics unit talks about the required process for mice that are occasionally sent into that place:

*“… we would expect […] a clean health screen before they even come anywhere near us. (…) I think they have to have at least six months to a year (…) so that we know that they’ve been clean for a couple of screens and then we put them, obviously, in our quarantine isolator (…). So they’re not just out on the shelf. We health screen them again just for our benefit and then once we know that they’re clean they will come out and we’ll put them into the correct rooms they go into.” (Ilana, NACWO, Lothlorien)*

The health status of an animal thus determines the ability for it to be received by another facility; and what constitutes assessing the health status of animals includes within it an assessment of the place that they come from. Moreover, this is a dynamic relationship, where animals potentially-exposed to infection lose their previous healthy status. For instance, animals that were bred and raised within a barrier facility^13^ and sent out to an airport but returned because of unsuitable temperatures for travel (see a discussion of environment, stress and travel in the next section) could not be returned to their original “clean” facility. Instead, they were kept in the quarantine suite as now they were considered “dirty”.^14^

Biosecurity, then, is a major determinant of the movement of animals because only those which can meet requirements gain passage. Where animals’ health or microbial statuses do not meet strict requirements set by certain facilities, the live animals themselves cannot enter. Yet, as we will further discuss in Sect. 4.2, the mobility of the strain can still be achieved through the transferral of animal body parts or embryos instead.

#### Welfare

Welfare, too, is an important factor shaping the regulation and practice of laboratory animals’ movement. As noted in Sect. 2, there is growing evidence for the effects of transport on laboratory animals’ experience. In turn, these changes threaten not only the animals’ welfare but also the outcomes of experiments themselves. As Jagger, a researcher, puts it;

*“if you’re bringing an animal over [from overseas] it’s massively stressful on them. You don’t want to use stressed animals for your experiment, you want them to be as nice and comfortable as possible”. (Jagger, University Researcher)*

Consequently, live animals’ mobilities are also shaped by the ways in which carers and transporters seek to keep them ‘as nice and comfortable as possible’.

Transport stress is often attributed to the affective atmosphere (Anderson, 2009; Lorimer et al., 2017) around the mouse changing, with no possibility of return to the baseline (Obernier & Baldwin, 2006, p. 365). The affective atmospheric changes can include changes in air temperature, smells, air flow, and how food and water accessibility may be altered by the change of environment; these changes whilst bringing a physiological impact to an animal, also can be physiologically read as a change in mood. The regulatory framework takes this into account when it requires that their atmosphere be managed in order to minimise stress. To that end, transport guidelines define minimum conditions in terms of food and water availability, space, and other specifications to seek to uphold a degree of familiarity in transport, as well as time to acclimatise.

Equipment and personnel are therefore deployed with a view to minimising the stress experienced by the animals. Mice are boxed in specially designed transport boxes for travelling (see Swallow et al., 2005 for specifications for different laboratory animal species). The boxes and materials travelling with the mice matter: after all, “material things are also bodies, influencing other bodies within their ambit, and being influenced in return” (Abram, 2011, p. 32). They, and the atmospheres they can engender within, are therefore critical to enabling animal (and animals’) mobility. For example, in Jorgensen’s account of muskox (*Ovibos moschatus)* relocation, the box carrying the animal is recognised to have material agency. She highlights its role in “creat[ing] and shap[ing] the interactions between the animal, humans, and the surroundings, putting the container into the role of the mediator” (after Latour) – and being able to “transform, translate, distort, and modify the meaning or the elements they are supposed to carry” (Latour, 2005, 39; in Jørgensen 2016, 100). Moreover, animals are transported via specialised couriers, who should be trained to deal with the special needs of their passengers or travel with someone who is (Swallow, 2005) - indeed, in one case, we heard of ex-Animal Technologists working as couriers. Couriers are required to keep track of the welfare of the animals in transit, but we found the transiting animal’s welfare also remained a concern for the sender until they reached their destination.

Expert knowledge of species-specific indicators of stress shapes observations of species during travel. It is this sort of attunement that enables animal care staff such as Lucas, below, to gauge the stress signs of different animals arriving from commercial suppliers. He notes that

*“The only time we ever see [signs of stress] is if we ever have rats. Rats can display that they’re stressed with porphyrin staining which looks like blood-stained tears. It’s a natural occurring thing in the animal. I’ve seen that quite a few times with rats which have come in when they’re unpackaged, [and] put to bed. And then an hour later somebody will check them and they’ll come back and they’ll say there’s blood, when in actual fact it’s porphyrin staining. And it’s just a mark that the animal is a little bit stressed. (…) that normally disappears.” (Lucas, NACWO, Lothlorien)*

Physiological measurements are also used to detect stress in moving animals (Obernier & Baldwin, 2006). For example, corticosterone has been found to be raised in blood samples of mice that had been moved within a facility (Drozdowicz et al., 1990). There is growing recognition that animal stress from transport can materially change the qualities of animal tissue and shape the reliability of experimental data and their extrapolation to humans (Bailey, 2018).

Another facet to animals’ mobilities is how their experience of travel depends on their companions. Those that are strange to each other cannot safely travel together, as fighting can result - nor can animals that travel alone be housed together. Either circumstance can lead to unexpected expenses for researchers. In the example below, for instance, a researcher ordered a batch of mice, assumed they could be group-housed in transit and on arrival, but in fact they all arrived in separate boxes and had to be housed separately at greater expense.

*“Animals have been delivered, and they’ve been [travelling] in individual boxes and in very small groups. ..then you’re not able to pool them, to make it that one big group cohort because you don’t know the origin of these animals, they could end up fighting.*

*(…) But it costs the user then more money ‘cause they’re having to put them in more cages when they come on site. So even though we’ve gone back to the suppliers and said, you know, when we’re asking for large cohorts like that in groups of ten for example can they be littermates, can they come from the cage that they were weaned in. But that sometimes falls on deaf ears, or whatever the process is, […] which costs the user more money. (Lydia, Chief Technician, interview)*

Communication between receivers of laboratory animals and their suppliers has been described to us as an important action in negotiating the practices of transportation. For instance, below, a laboratory vet explains how:

*“…the commercial suppliers have changed their attitude on how to put animals together in a transport box, because in the past they were randomly put in there and they had so many issues with fighting”. (Gretchen, Named Veterinary Surgeon at a university)*

The communication between suppliers and recipients is a source of iteration and adaptation in practice as more is learnt, or tested, to see if it improves the travel experience for the mouse.

Welfare, then, is an ever-present concern that shapes how animals are made mobile. Although here we have dealt especially with practices of welfare protection, it bears noting that other normative questions regarding the geographies of mouse welfare remain unanswered; that is, it is easier to get assurances about their status during travel than what comes afterwards, as illustrated by this reflection from Lindsey, an animal technician:

*“She wonders sometimes about what happens to the mice after they are sent out to other places, and specifically their welfare. […] Are there places that they should not send them to because their welfare may not be assured? She says that they remain responsible for the mice that are sent out to customers in terms of their welfare [only] during transport.” (Field notes from participant observation with Lyndsey, August 2018).*

### Embryo and gamete mobilities

When the live animals are not considered of high enough health status or the concerns for the welfare of the animal(s) during transit are too significant, the strain can still move. Embryos then enter our story as a material form that can make a mouse strain mobile, via processes such as re-derivation. Rederivation involves the practice of sacrificing a pregnant mouse, and implanting her embryos that are first “cleaned” in the laboratory into a foster mother with a higher health status:

*Everything that goes into [specific pathogen free facilities] has to be re-derived so we would never put mice straight into there from any source. And the same with all the other mice that we source, they all have to be re-derived because we wouldn’t bring a live mouse from another university, institution or whatever, into our animal facility. So yeah, I guess it is really reputational […] but we don’t generally use anyone else other than these two suppliers [for live animals], or they are re-derived [before entering]. (Jeremy, Operations, university)*

Where animals themselves cannot circulate, rederivation thus enables strains to be “cleaned” and enter a space. Mobility becomes possible by harnessing the capacity of the animal body to reproduce and create embryos. Jagger tells of a time when they wanted to move their mice into a brand new building (see also Fray et al., 2008 for a discussion of re-derivation as having health and welfare benefits):

*I mean in my old place we were changing mouse houses, so we built a (…) brand spanking new place and we needed to transfer mice over from one unit to another. But, you can’t actually introduce mice so you have to–, we had to, sort of, en masse [yeah] use [a large commercial breeding company] in that case to freeze down embryos and transfer embryos.” (Jagger, lines 923–930)*

Through this process of re-derivation, embryos become the proxy (Parry, 2004, p. 74) that is mobilised to enter the “clean” space. Through this purifying decorporealisation, researchers can import the strain and make it acceptable to that place. That such a system exists in the first place speaks, of course, to the emergent nature of the colonies of mice.

Embryos also have an increasing role to play as an alternative to the transport of live animals – especially in the case of genetically altered animals (Swallow et al., 2005; RSPCA Resource Sharing Working Group 2009; Mahabir et al., 2008; Kelley, 2010; Robinson et al., 2003, p. s1:38). Joint reports from animal welfare organisations regarding the welfare of genetically altered (GA) animals have repeatedly recommended embryo or gamete transport instead of live animals in recognition that transporting live mice causes stress in the animals, and this suffering may be compounded in the case of genetically altered animals, who may be in ill health (Robinson et al., 2003; RSPCA Resource Sharing Working Group 2009). This recommendation is also part of the guidance produced by LASA’s Transport Working Group (Swallow et al., 2005, p. 15).

Whereas commercially available inbred strains can be routinely obtained as live animals through the practices described in previous sections, these guidelines link the particular ontology of GA animals to the need for different standards of travel. Their experience of being passengers could cause too much harm. Hence, it illustrates again how their mobilities, albeit fully determined by the humans responsible for their care, take animals’ affective experience into account. This is not to say that welfare is the only value being taken into consideration. Importing embryos is also considered to be more biosecure, when compared to importing live animals, reducing the risk of introducing infections into the animal house. As such, transporting embryos or gametes can provide an alternative means of mobilising a line that bypasses the health and welfare issues we detailed earlier in the paper. However it does require the receiving facility to be able to carry out the artificial reproductive procedures required to encourage their development into animals (Du et al., 2010).

At one of our ethnographic fieldwork sites, this replacement of live animal transport with shipping embryos or gamete tissue from which to extract semen, was an important strand of their welfare-oriented work. The lack of recognised sentience of the embryo, together with its capacities for reproduction, make it a suitable stand-in for the whole grown animal, even if it has considerable differences in terms of the timescales for having the animals be “ready” for experimental procedures. The shipping of gametes instead of live animals provides a simpler and cheaper alternative, in addition to its welfare gains – although it does require biotechnological capabilities at the receiving site. We found considerable efforts made to innovate ways of transporting sperm-containing body parts that require increasingly simple shipping containers and lower temperatures. This institution that shipped and received animals regularly sought to encourage others to avoid sending animals where possible. This was through the work of customer-facing staff members who disseminated information about this alternative and emphasised its economic and welfare advantages.^15^

Different values are emerging as the embodied capacities and experiences of sentient animals and their non-sentient replacements are meaningfully enrolled into the governance of animal mobilities. A different type of strain mobility is emerging: that of transporting non-sentient forms of the embodied strain. Correspondingly, there is a considerably lighter regulatory “burden” and financial burden when transporting embryos and mouse body parts, as opposed to live animals, for example we heard of live animal freight charges around the sum of “£1400”^16^. Our empirical findings suggest, that the very latency of the embryo or gametes in a cryopolitical sense (Radin & Kowal, 2017), that life exists but is not yet developed, enables them to bypass concerns about sentience and infection that are very present in the ethico-onto-epistemological entanglement of transporting live animals.

## Discussion

We have described features of the contemporary UK animal research industry engaged with mice strain mobility which is both receiving and sending animals that have had or will embark upon, a journey of different lengths. It is a normalised practice, but one where there are ongoing care tinkerings (Mol and Law 2010) and significant biotechnological innovations that respond to changes in scientific, animal welfare and biosecurity regulatory and market concerns. These are found in the form of animal health-checks, mouse transport boxes, specialised strain care information, quarantine practices, material transfer agreements and other socio-technical practices that support animal movement – through to the replacement in some cases of transporting live animals with germinal products that are then rederived once in their new home. Whilst research animal transport is often secretive, this papers sheds light on socio-technical and cultural changes that are informing the scientific expectations about travelling animal models and the animal care that supports it.

The two forms in which the strain travels either as live sentient animals or as non-sentient, non-living embryos and gametes have different mobilities. As we have shown, their different mobilities are shaped by how their form relates to different regulatory, capital, ethical and scientific values attached to sentience and biosecurity and commodity-availability. Recognising this emphasises that whilst the research model can be an exchangeable-commodity, it is not an alien commodity object as we have shown that values surrounding it affect how and where it is sold from and who can buy it or access it (Tsing, 2015). These values are made through the practices of animal care technicians, scientists and other mediators who make the strain valuable in ways that cohabit with capitalism. When the strain is gifted, shared, bought, and sold it therefore perpetuates the making of values associated with it as both a sentient living animal and as a scientific tool, yet never solely its capital exchange value. These findings resonate with arguments that feminist philosophers of science have been making about ‘situated knowledges’ (Haraway 1998) and ‘diffraction’ (Barad 2006) in terms of how the processes that surround and embody the experience of mobility inescapably result in knowledges changing whilst moving through sites, also referred to as material diffraction. And one could argue it is through laboratory animal strain mobilities and strains’ mobilities that creative opportunities to do and know things otherwise, rather than representing universal consistency, are realised as cultures meet, technology travels, knowledge is exchanged not only scientific but also around animal welfare and biosecurity expectations. And yet whilst aspirations exist for a mouse common where strains are freely-exchanged, it is also obvious how exclusionary scientific infrastructures that maintain high-level biosecurity and address negative animal welfare associated with travel, most closely, for example rederivation facilities, do create new infrastructural barriers to strain access. These changing cultural economic geographies of mouse strain mobilities and mice experiences of mobility leads us to speculate about future potential changes. In part this is to demonstrate the current contingency of the ethico-onto-epistemological arrangement we describe, but also to challenge current assumptions about who moves in laboratory animal research, and what the experimental encounter is like.

Imagine a world where the laboratory animal strain never moved and instead the researchers travelled to the animals they wanted to work with. In other words, researchers with Health Status reports had to spend time doing weeks or months of fieldwork in the laboratory home of their animal of interest. This we learnt would still not curtail the need for some animals or their body parts to travel, since fresh breed-stock is necessarily acquired from elsewhere to maintain the genetic line; yet it would significantly reduce the numbers of animal travelling and more particularly address concerns that transported animals need time to recover before experimentation, or the time for rederivation processes to be completed. The outcome would be that the various costs of travel would be distributed differently across humans and animals in the animal research nexus (Davies et al. 2020), and here a different power dynamic between humans and animals could shape animal mobilities and animals’ mobilities (Hodgkin and Lorimer 2015). Then, imagine another world; where the experimental encounter between researchers and mice could take place remotely thus reducing the need to meet face-to-face, but still with sufficient material intimacy that experiments could be ran and data collected. For some senior researchers who have a team performing the day-to-day experiments this is not far from the current reality. In both worlds the field site would include a team of animal care technicians with specialised expertise in the strain to support the visiting or virtual researcher to execute the experiments planned during their period of fieldwork. There could be the opportunity to mingle and collaborate with other researchers working on the same strains through virtual or face-to-face co-location. These speculative narratives draw attention to - and therefore highlight the contingency of the contemporary ‘ethico-onto-epistemological entanglement’ (Barad, 2014) that we have described in this paper. And they create space for questions about which courses of action are available as what shapes the contemporary entanglement becomes clearer through our analysis (Giraud, 2019); what further adaptations and innovations maybe witnessed and understood in relation to future changes in values, practices and animal and human experiences?

Against a backdrop of public concern about the welfare of travelling animals more generally, it is evident that the political and everyday cultures around human-animal relations across different animal species in different human social worlds continues to deepen awareness about the implications of animal sentience when transporting animals for research. Animal care technicians and the associated changes in the culture around their work, have an important role here, as they are encouraged to speak-out about animal care concerns, which once were marginalised from shaping scientific practices. The recent regulatory turn in supporting a ‘culture of care’ (Boden & Hawkins, 2016) supports attentiveness to the mouse as sensitive and vulnerable to changes related to travel experiences. This, along with care technicians’ personal experiences of laboratory biosecurity failures, leading to sick animals and mass animal culls, sensitises the industry to the various risks associated with the exchanges of animal strains - standardised animal research models – between research teams. Trust in health documentation accompanying an animal is critical, but the advent of opportunities to avoid laboratory animal movement again eases this process as less is at stake. Consequently, replacing a transport box of warm living animals with a tube of frozen suspended-life is becoming ever more common and deemed best practice where practicable.

## Footnotes

1 This project received ethical approval from [REDACTED FOR BLINDING].

2 NC3Rs: Best practice for animal transport. https://nc3rs.org.uk/best-practice-animal-transport Accessed 29/07/2021.

3 Leonard, interview. See also AnimalRightsExtremism.Info: *Groups and Campaigns Overview.*http://www.animalrightsextremism.info/animal-rights-extremism/existing-groups-campaigns/groups-campaigns-overview/ Accessed 07.08.20. The original website www.gatewaytohell.net is now inactive.

4 See ‘Model Health Certificate for Oher Rabies Susceptible Species (including Rodents, Lagomorphs, Carnivores and other Mammals) OTH GBHC175E v1.0’, available online at https://assets.publishing.service.gov.uk/government/uploads/system/uploads/attachment_data/file/975839/Other_rabies_susceptible_species_from_the_EU_OTH_GBHC175E.pdf. Accessed 28.05.21.

5 UK Government. Guidance: Moving live animals or animal products as part of EU trade. Published 12 Sep 2014, last updated 20 Aug 2019. https://www.gov.uk/guidance/moving-live-animals-or-animal-products-as-part-of-eu-trade#display-laboratory-and-research-animals/. Accessed 16.04.2020.

6 A description of the UK system of controls on import of live animals and animal products and evaluation of its performance to protect public and animal health (April 2016-March 2017). Presented to Parliament pursuant to section 10 A of the Animal Health Act (as amended by the Animal Health Act 2002) September 2018. p. 5 Available online at https://www.assembly.wales/laid%20documents/gen-ld11725/gen-ld11725-e.pdf.

7 A description of the UK system of controls on import of live animals and animal products and evaluation of its performance to protect public and animal health (April 2016-March 2017). Presented to Parliament pursuant to section 10 A of the Animal Health Act (as amended by the Animal Health Act 2002) September 2018. p. 5 Available online at https://www.assembly.wales/laid%20documents/gen-ld11725/gen-ld11725-e.pdf.

8 The European Council Regulation (EC) No. 1/2005 sets out the rules for all live vertebrate transport for economic purposes, which is further enshrined in UK law (via The Welfare of Animals (Transport) (England) Order 2006 and similar legislation for Scotland, Wales and Northern Ireland). The European Convention for the Protection of Animals during International Transport (Revised) (ETS/193) also applies. Air travel is regulated by the International Air Travel Association (IATA).

9 NC3Rs. Best Practice for Animal Transport. https://www.nc3rs.org.uk/best-practice-animal-transport. Accessed 07.08.2020.

10 Different authors refer to it differently – e.g. as the Mouse Academic Research Commons (Einhorn & Heimes, 2009) or as the Mouse Academic Commons (Parry, 2019), or a “biomedical research commons” (e.g. Bubela et al., 2017).

11 Field notes, 31st of July 2018.

12 Interview Isabelle, administrative staff at a University.

13 These are facilities where “comprehensive programmes for the prevention of pathogen contamination” are implemented, with standard operating procedures setting out a variety of criteria for environmental systems, animal handling and housing, and entry of people, materials and animals through the barrier (Institute of Laboratory Animal Resources 2000, p. 17).

14 Field Notes, August 2018.

15 Interview, Kelly.

16 Interview, Jacqueline.
